# Anillin-dependent organization of septin filaments promotes intercellular bridge elongation and Chmp4B targeting to the abscission site

**DOI:** 10.1098/rsob.130190

**Published:** 2014-01-22

**Authors:** Matthew J. Renshaw, Jinghe Liu, Brigitte D. Lavoie, Andrew Wilde

**Affiliations:** 1 Department of Molecular Genetics, University of Toronto, 1 King's College Circle, Toronto, Ontario, CanadaM5S 1A8; 2 Department of Biochemistry, University of Toronto, 1 King's College Circle, Toronto, Ontario, CanadaM5S 1A8

**Keywords:** cytokinesis, cell division, mitosis, high-resolution microscopy

## Abstract

The final step of cytokinesis is abscission when the intercellular bridge (ICB) linking the two new daughter cells is broken. Correct construction of the ICB is crucial for the assembly of factors involved in abscission, a failure in which results in aneuploidy. Using live imaging and subdiffraction microscopy, we identify new anillin–septin cytoskeleton-dependent stages in ICB formation and maturation. We show that after the formation of an initial ICB, septin filaments drive ICB elongation during which tubules containing anillin–septin rings are extruded from the ICB. Septins then generate sites of further constriction within the mature ICB from which they are subsequently removed. The action of the anillin–septin complex during ICB maturation also primes the ICB for the future assembly of the ESCRT III component Chmp4B at the abscission site. These studies suggest that the sequential action of distinct contractile machineries coordinates the formation of the abscission site and the successful completion of cytokinesis.

## 2. Introduction

At the end of each cell division cycle, a mother cell is physically cleaved into two new daughter cells, each containing a complete copy of the genome [1,2]. This process of cytokinesis is essential for the propagation of cells and the development of an organism. Failure to successfully coordinate segregation of the genetic material with the physical act of cell division leads to genetic instability and aneuploidy [3,4].

In animal cells, the actomyosin cytoskeleton generates a contractile force to invaginate a furrow of plasma membrane between the segregating chromosomal masses during anaphase [5]. The positional cue for the site of membrane invagination comes from signals delivered by the microtubule cytoskeleton [6,7]. During invagination, the actomyosin contractile ring must remain attached to the plasma membrane and be stabilized within the central region of the cell. Anillin, a multidomain protein with the capacity to link many elements of the contractile machinery, including the plasma membrane and the actomyosin and septin cytoskeletons [8], contributes to the attachment and stabilization of the contractile ring during furrow ingression [9,10]. Disruption of any of these factors causes early defects in cytokinesis by disrupting furrow stability and ingression [5].

As furrow ingression proceeds, the microtubules of the central spindle become increasingly bundled and become ensheathed in a tube of plasma membrane called the intercellular bridge (ICB), the final connection between the two newly forming daughter cells. Microtubule bundling is independent of furrow ingression; however, it is not known whether the ICB forms passively around the bundled microtubules or through an active process to make the membranous tube. The ICB is an important structure and it is within the ICB that abscission occurs [11,12]. The ICB is a highly dynamic structure that is remodelled as it matures to form an abscission site within itself [13]. Indeed, many factors required for abscission are first targeted to the stem body, the central bulge of the ICB, before relocalizing to the abscission site [13–16]. When an ICB fails to form, no site of abscission can be generated and cytokinesis fails, resulting in multinucleate cells [17]. Interestingly, the site of abscission forms to one side of the central stem body. The endosomal sorting complex required for transport III (ESCRT III) machinery, which is required to execute abscission [14,18], is first recruited to the stem body before relocalizing to a constriction site formed to one side of the stem body and marking where the final act of abscission occurs [15,16]. How this constriction site forms remains to be determined.

In mammalian cells, anillin is required for the early stages of cytokinesis to stabilize the furrow and recruit septins to the cytokinetic machinery [9,10]. However, both anillin and septins remain part of the ICB after the furrow has ingressed, suggesting they may have roles in cytokinesis beyond regulating furrow ingression [10,19]. Consistent with this, the early role of anillin in cytokinesis in *Drosophila* S2 cells can be bypassed, and the cells consequently show defects in midbody ring assembly [20–22]. Likewise, in mammalian cells, depletion of SEPT9 prolongs cytokinesis with an eventual failure to abscise [23]. These combined observations suggest that anillin and septins may have specific functions during the later stages of mammalian cytokinesis.

Although recent studies have shed light on the final stages of abscission and its regulation [14–16,24,25], little is known about the mechanism of ICB formation that provides the platform for subsequent cell abscission. Here, we investigate the role of anillin in ICB formation and maturation through its function in recruiting the septin cytoskeleton. Using novel tools that allow initial furrow ingression to progress in the absence of the septin cytoskeleton combined with live imaging and subdiffraction three-dimensional structured illumination microscopy (3D-SIM), we define new stages and structures required for ICB assembly and abscission in mammalian cells.

## 3. Results

### 3.1. Anillin dynamically associates with late cytokinetic structures

**Figure 1.**
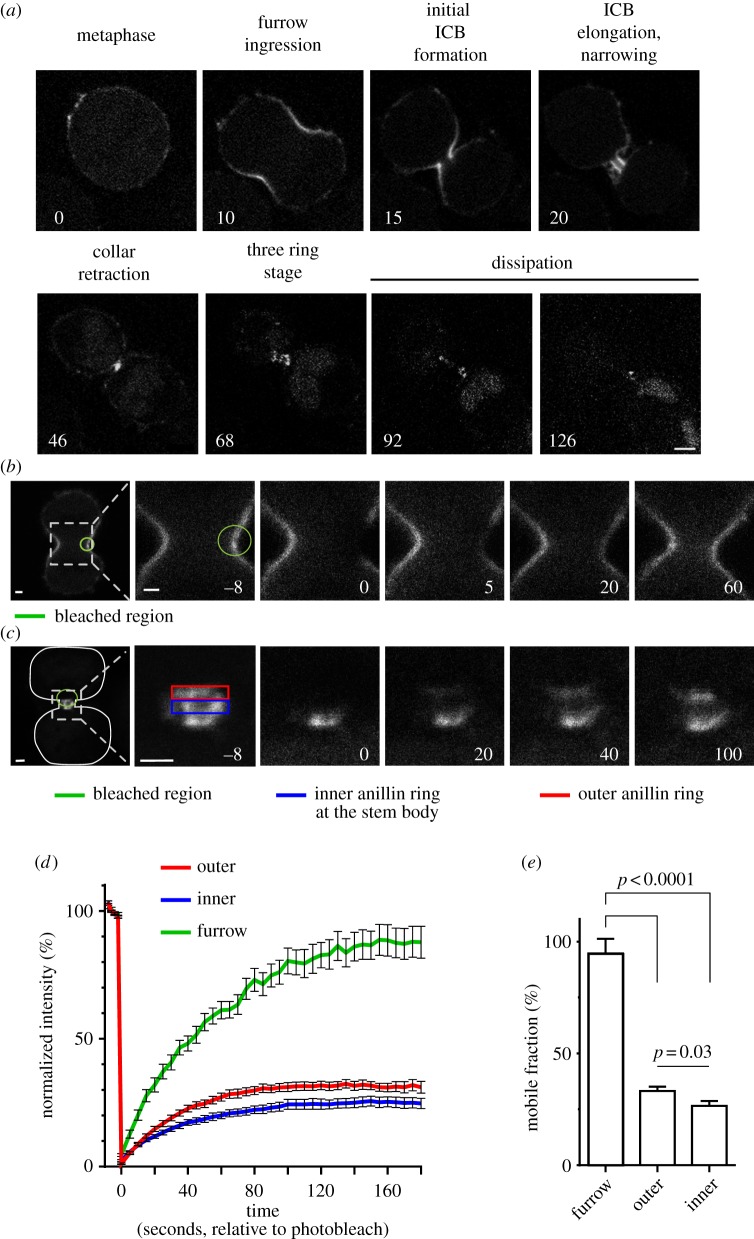
Anillin dynamics during cytokinesis. (*a*) Micrographs from a time-lapse series taken of HeLa cells expressing GFP-anillin from the point of entry into anaphase through to abscission. Numbers are minutes from the onset of anaphase. Scale bar, 5 μm. (*b*) Micrographs from a time-lapse series taken of a HeLa cell expressing GFP-anillin before and after a region (green circle) of the plasma membrane in the furrow was bleached. The first panel shows both forming daughter cells, and the subsequent panels are a magnification of the boxed region of the first panel. Scale bar, 2 μm. (*c*) Micrographs from a time-lapse series taken of a HeLa cell expressing GFP-anillin before and after a region (green circle) of the ICB encompassing the central anillin ring (blue rectangle) and one anillin ring at a constriction site (red rectangle) were bleached. The first panel shows both forming daughter cells, and the subsequent panels are a magnification of the boxed region of the first panel. Scale bar, 2 μm. (*d*) Graph following the recovery of GFP-anillin fluorescence after the photobleaching described in (*b,c*). (*e*) Comparison of the mobile fractions of GFP-anillin in each region. Mean ± s.e.m. is shown.

To assess anillin's role in the late stages of cytokinesis, we analysed anillin dynamics during cytokinesis in a HeLa cell line stably expressing inducible green fluorescent protein (GFP)-anillin at levels similar to endogenous anillin (see electronic supplementary material, figure S1). Time-lapse analysis of GFP-anillin revealed previously undescribed phases of anillin organization, suggesting new distinct stages of cytokinesis where anillin may function (figure 1*a* and the electronic supplementary material, video S1). Prior to chromosome segregation, anillin is distributed along the plasma membrane before it concentrates in the furrow upon ingression. As the opposing membranes of the furrow converge, anillin localizes to a distinct collar that defines the initial ICB (1.89 ± 0.08 μm diameter, and 1.27 ± 0.03 μm long, as measured along the axis of the ICB, *n* = 18). Next, the anillin collar elongates almost threefold to 3.61 ± 0.25 μm in length and narrows by 25% to a diameter of 1.44 ± 0.06 μm, *n* = 18 (see electronic supplementary material, video S2). The anillin collar then retracts and reorganizes to form three distinct rings (figure 1*a* and the electronic supplementary material, figure S2*a,b*); the central ring lies within the stem body, and the two flanking rings have a narrower diameter (see electronic supplementary material, figure S2*a,b*). Subsequently, anillin leaves the ICB. Throughout these stages, anillin co-localized with septins (see electronic supplementary material, figure S2*b*).

To assess the stability of anillin within the different structures, we analysed anillin dynamics by fluorescence recovery after photobleaching (FRAP; figure 1*b–e*). When GFP-anillin was bleached in the regions of the ingressing furrow, the GFP-anillin signal recovered fully (94.7 ± 6.6%, *t*_1/2_ = 48.2 ± 4.6 s), suggesting there is a rapid turnover of anillin either within the plane of the membrane or between the membrane and the cytosol (figure 1*b,d,e* and the electronic supplementary material, video S3). By contrast, GFP-anillin localized to the ICB later in cytokinesis showed lower levels of recovery after photobleaching (figure 1*c–e* and electronic supplementary material, video S4). Later in cytokinesis at the three-ring stage, GFP-anillin was bleached at the central stem body and one constriction site. Now, only 33.2 ± 1.9% of GFP-anillin signal at the constriction site was recovered. However, this was significantly more than the 26.5 ± 2.2% of the GFP-anillin signal recovered at the stem body (*p* = 0.03, figure 1*c–e*). These data suggest that as cytokinesis progresses anillin becomes increasingly stable within the structures it resides.

**Figure 2.**
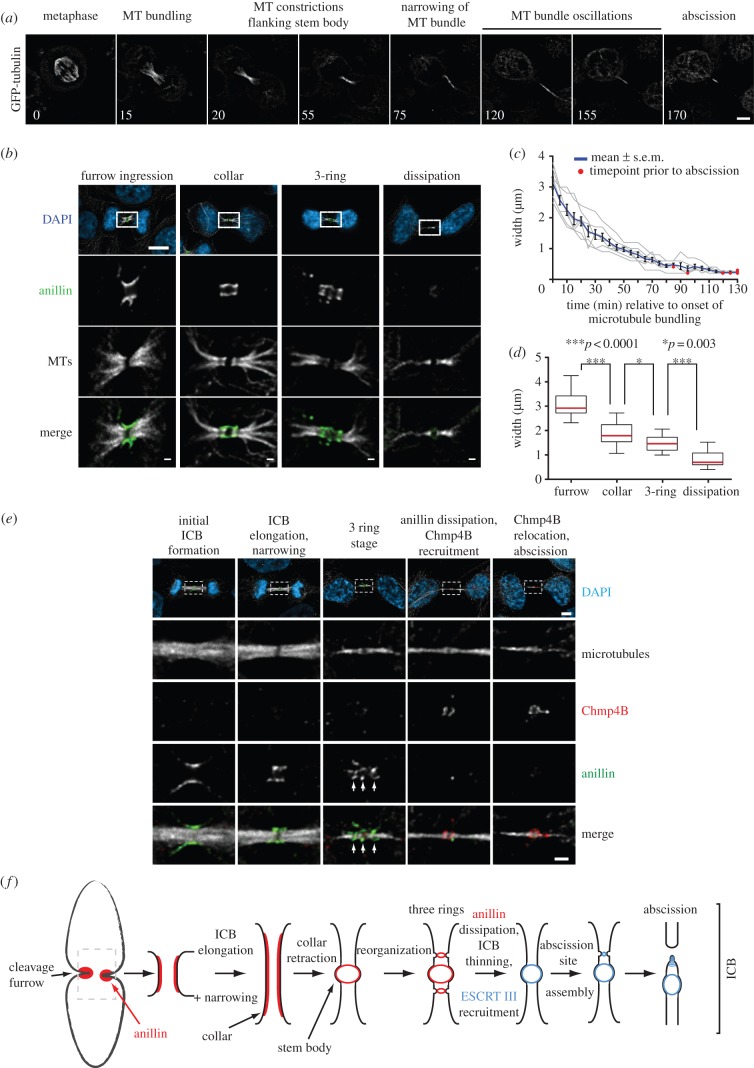
The ICB forms through a series of defined organizational states. (*a*) Micrographs from a time-lapse series taken of HeLa cells expressing GFP-tubulin from the point of entry into anaphase through to abscission. Numbers are minutes from the onset of anaphase. Scale bar, 5 μm. (*b*) Micrographs of fixed HeLa cells stained with anti-anillin and anti-tubulin antibodies. Scale bar, 5 μm in whole cell images and 1 μm in magnified images. (*c*) Measurement of the width of the microtubule bundle in the ICB overtime. Grey lines are traces from individual cells, the blue line the average. (*d*) ICB microtubule width during different anillin organization states. Furrow, *n* = 17, collar, *n* = 28, three-ring, *n* = 15 and dissipation, *n* = 38. Red line is the median and the boxes mark the 25th–75th percentile range. (*e*) Stably expressing GFP-tubulin HeLa cells were fixed and stained with anti-anillin and anti-Chmp4B antibodies. The organizational state of GFP-microtubules in fixed cells was compared with that observed in live imaging, (*b*), to order the ICBs in increasing states of maturity from left to right. White arrows point to the three anillin rings. Scale bar, 5 μm in whole cell images and 1 μm in magnified images. (*f*) Schematic outlining the different stages of anillin (red) and ESCRT III (blue) organization during cytokinesis. MT, microtubule.

To further assess anillin dynamics, we developed a method to stage ICB maturity by correlating the anillin organizational state with that of the microtubules, a marker present throughout all stages of ICB maturation. We analysed microtubule organization during ICB assembly until abscission in GFP-tubulin expressing HeLa cells (figure 2*a* and the electronic supplementary material, video S5). The microtubules of the spindle midzone become increasingly bundled as the furrow ingresses. As the microtubule bundles become denser, two regions of lower GFP-tubulin intensity form on either side of the central bulge that marks the stem body. We refer to these regions of decreased microtubule staining as constriction sites because they are of a narrower diameter than the microtubule bundles elsewhere in the ICB. Next, the microtubule bundle narrows further to the diameter of the two constriction sites, resulting in a bundle of microtubules with a uniform diameter, except for a less pronounced bulge of GFP-tubulin intensity at the stem body. Following this, we observed the oscillation of the microtubule bundle from side to side within the ICB. Finally, asymmetric cleavage occurs, termed abscission, and the ICB remnant is consumed by one of the daughter cells.

As the ICB forms and cytokinesis progresses, the microtubule bundle undergoes a process of thinning as cytokinesis progresses (figure 2*a*). We used the width of the microtubule bundle as a reference point for ICB maturity (figure 2*c*). To further dissect the different states of ICB maturation, we correlated the thickness of the ICB microtubule bundles for each category of anillin localization. Consistent with the observations from live cell imaging, analysis of fixed cells stained for anillin and tubulin (figure 2*b*) demonstrated that the progression through the distinct stages of anillin organization correlated with defined windows of microtubule bundle width (figure 2*d*), allowing the maturity of individual ICBs in fixed cells to be determined, thereby allowing ICBs of similar maturity to be compared.

As we observed anillin leaving the ICB, we determined at what stage of cytokinesis this happened. Anillin had previously been suggested to have a direct role in abscission [19]. To relate anillin dynamics to abscission in fixed cells, we co-stained GFP-tubulin expressing cells with antibodies recognizing anillin and Chmp4B, an ESCRT III component directly involved in abscission [14]. We confirmed previous observations that Chmp4B is first recruited to the stem body before moving to the abscission site [14]. Interestingly, Chmp4B only begins to enter the ICB as anillin leaves (figure 2*e*). These data suggest that anillin and the septins have no direct role in abscission. Our observations thus define ICB elongation and narrowing as preceding constriction site formation, all of which occur before the recruitment of the ESCRT III component Chmp4B and the assembly of the abscission site. These new discrete steps in ICB formation and maturation suggest new, hitherto unappreciated roles for anillin and septins (figure 2*f*).

### 3.2. Septin recruitment to the furrow increases ingression efficiency

We next analysed cytokinesis in the absence of septin recruitment to the cytokinetic machinery. Owing to the functional redundancy of many individual septins, we devised an experimental strategy to eliminate all septin recruitment to the cytokinetic furrow without disrupting non-mitotic septin functions. We expressed a chimeric anillin (anillinΔPH-PLCδPH) where its PI(4,5)P_2_ and septin-binding PH domain was replaced with the PH domain of PLCδ1, which binds PI(4,5)P_2_ but not septins [10] (see electronic supplementary material, figure S3*a,b*). Simultaneously, endogenous anillin was depleted by siRNA targeted to the 3′UTR without disrupting the translation of ectopically induced, siRNA-resistant, GFP-anillin or GFP-anillinΔPH-PLCδPH (see electronic supplementary material, figure S1). As expected, GFP-anillinΔPH-PLCδPH failed to recruit septins to the cytokinetic machinery when expressed as the sole form of anillin in cells [10]. By contrast, RhoA, activated myosin and actin were recruited to the early cytokinetic structures in cells expressing anillinΔPH-PLCδPH as the only form of anillin, in a manner indistinguishable from cells only expressing GFP-anillin (see electronic supplementary material, figure S3*c–e*; Liu *et al*. [10]). In addition, the profile of proteins that co-purified with immunoprecipitation of GFP-anillin or GFP-anillinΔPH-PLCδPH was indistinguishable (see electronic supplementary material, figure S3*f*), suggesting that as far as we can examine GFP-anillin and GFP-anillinΔPH-PLCδPH interact with the same cohort of factors with the exception of the septins. Furthermore, while anillinΔPH-PLCδPH supported the formation of correctly positioned symmetric furrows [10], these cells failed to complete cytokinesis successfully (see electronic supplementary material, video S6). These data indicate that anillin-mediated septin recruitment to the cytokinetic machinery is required in the late stages of cytokinesis.

To investigate the role of anillin-mediated septin recruitment in the final stages of cytokinesis, we characterized the phenotypes of cells expressing GFP-anillinΔPH-PLCδPH as the sole source of anillin. Although cleavage furrows were both symmetrical and located in the centre of the cell, as seen in wild-type or cells expressing GFP-anillin [10], we observed differences in the kinetics of furrow ingression. In all cells, we observed two phases on furrow ingression: a slow initial phase followed by a rapid second phase (see electronic supplementary material, figure S4*b,c*). Both parameters were about 50% in cells expressing GFP-anillinΔPH-PLCδPH (see electronic supplementary material, figure S4*b,c*). In addition, the slow phase in GFP-anillinΔPH-PLCδPH expressing cells persisted twice as long as cells expressing GFP-anillin (see electronic supplementary material, figure S4*d*). However, although slower, GFP-anillinΔPH-PLCδPH expressing cells did complete furrow ingression. By contrast, GFP-anillinΔPH-PLCδPH expressing cells failed to complete many of the later events in cytokinesis, including ICB elongation and narrowing, the extrusion of tubules from the ICB and generation of microtubule constriction sites and abscission site (see below).

### 3.3. Anillin-dependent recruitment of septins promotes intercellular bridge formation

**Figure 3.**
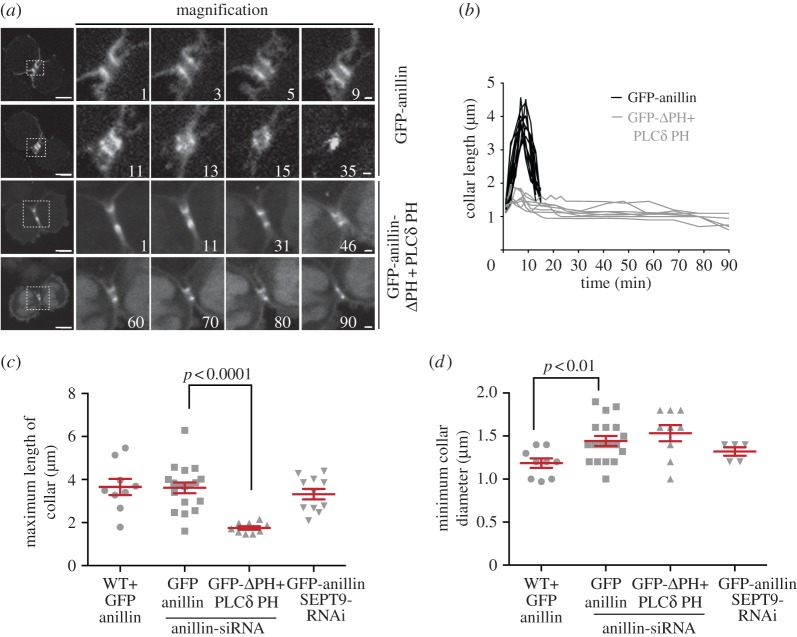
Anillin-dependent recruitment of septins is required for ICB elongation. (*a*) Time-lapse series of HeLa cells expressing GFP-anillin or GFP-anillinΔPH + PLCδ PH as the only forms of cellular anillin during the ICB elongation phase of cytokinesis. Left-hand panel, full cell view, scale bar, 5 μm; right-hand panels, magnified views of the ICB, scale bar, 1 μm. Time 0 is the point of initial ICB formation. (*b*) Length of anillin–septin collar over time during the ICB elongation phase in HeLa cells expressing GFP-anillin (black, *n* = 12) or GFP-anillinΔPH + PLCδ PH (grey, *n* = 8) as the only forms of cellular anillin. (*c*) Histogram of maximum anillin ICB collar lengths in HeLa cells overexpressing GFP-anillin, WT + GFP-anillin, HeLa cells expressing GFP-anillin or GFP-anillinΔPH + PLCδ PH as the only forms of cellular anillin and GFP-anillin expressing HeLa cell depleted of SEPT9, GFP-anillin SEPT9 RNAi. (*d*) Histogram of the minimum diameter of ICBs in cells expressing different forms of anillin at different levels. WT, wild-type.

The earliest post-furrow ingression role for the septin cytoskeleton is in the elongation of the ICB (figure 3 and the electronic supplementary material, videos S2 and S7). While in GFP-anillin expressing cells the initial ICB collar elongates threefold (see electronic supplementary material, video S2), the collar in GFP-anillinΔPH-PLCδPH expressing cells barely elongates (see electronic supplementary material, video S7). Interestingly, the collar in GFP-anillinΔPH-PLCδPH expressing cells has dimensions similar to GFP-anillin expressing cells (diameter = 2.13 ± 0.11 μm, length = 1.29 ± 0.07 μm, *n* = 9); however, in GFP-anillinΔPH-PLCδPH, the collar only elongates to a maximum length of 1.75 ± 0.08 μm (*n* = 9), significantly less than in cells expressing GFP-anillin (*p* < 0.001), and narrows to 1.53 ± 0.09 μm in diameter (*n* = 9, figure 3*b–d*). In contrast, overexpression of GFP-anillin caused an increase in collar length, similar to GFP-anillin rescue cells (1.31 ± 0.05 to 3.65 ± 0.38 μm, *n* = 9, figure 3*c*), but a 50% reduction in collar width (2.28 ± 0.06 to 1.19 ± 0.06 μm, *p* < 0.01, *n* = 9, figure 3*d*), significantly greater than other conditions. Therefore, ICB collar elongation and narrowing is dependent on the septin recruitment potential of anillin.

### 3.4. Anillin–septin filaments form within the intercellular bridge

**Figure 4.**
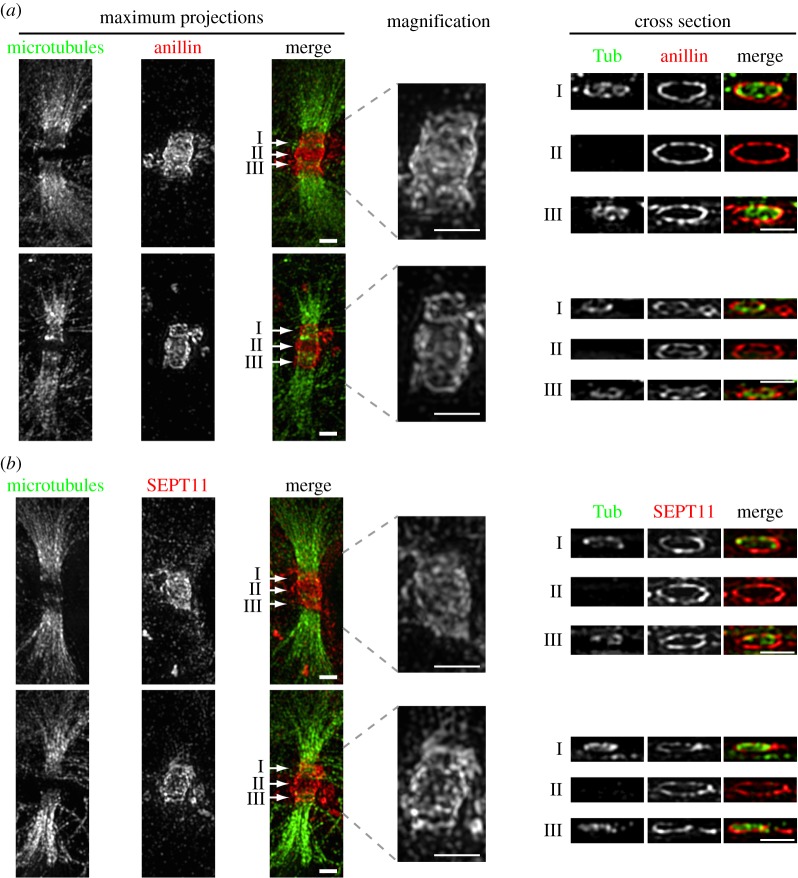
Subdiffraction microscopy images of anillin and septin organization in ICB during their elongation phase. (*a*) TCA fixed Hela cells stained with anti-tubulin (Tub) and anti-anillin antibodies. Left-hand panels are maximum projections, right-hand panels are cross section though different points of the ICB marked I, II and III. Scale bar, 1 μm. (*b*) TCA fixed HeLa cells stained with anti-tubulin and anti-SEPT 11 antibodies. Left-hand panels are maximum projections, right-hand panels are cross section though different points of the ICB marked I, II and III. Scale bar, 1 μm.

**Figure 5.**
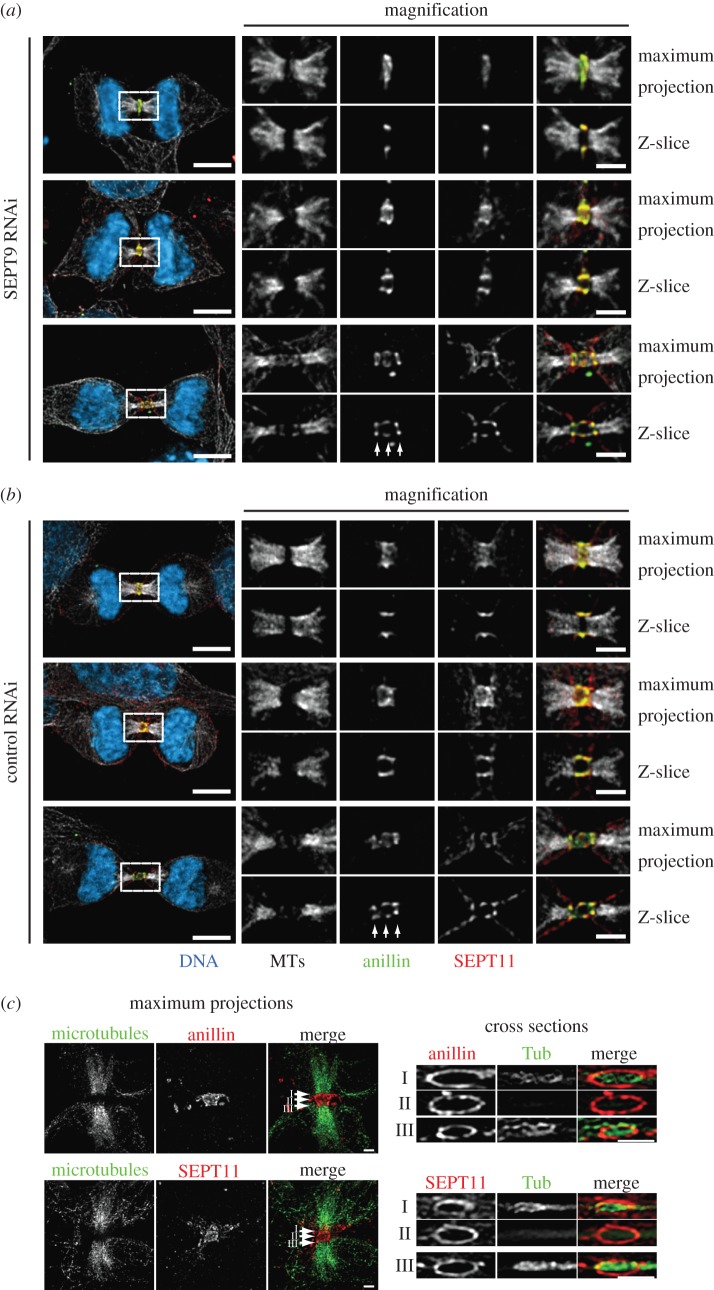
SEPT9 is not required for ICB formation. (*a*) Localization of anillin and SEPT11 in the absence of SEPT9 in ICBs of increasing maturity. White arrows point to the three discrete rings of the three-ring stage of anillin organization. Scale bar, 5 μm. (*b*) Localization of anillin and SEPT11 in absence of endogenous SEPT9 in ICBs of increasing maturity. White arrows point to the three discrete rings of the three-ring stage of anillin organization. Scale bar, 5 μm. (*c*) 3D-SIM images of TCA fixed Hela cells transfected with SEPT9 RNAi stained with anti-tubulin and anti-anillin or anti-SEPT11 antibodies. Left-hand panels are maximum projections, right-hand panels are cross section though different points of the ICB marked I, II and III. Scale bar, 5 μm.

To further characterize the elongation process, we next visualized the anillin–septin cytoskeleton within the ICB using 3D-SIM [26–29]. We observed ring-like filamentous anillin–septin arrays perpendicular to the long axis of the ICB (figure 4 and the electronic supplementary material, videos S8 and S9). By using 3D-SIM, we could not distinguish whether septins form a long continuous helical filament or a series of shorter circular filaments. To try to resolve whether septins are required to form long or short filaments for ICB elongation, we assessed the role of SEPT9 in ICB elongation and septin filament formation. SEPT9 is required for the formation of long septin filaments in interphase and the successful completion of cytokinesis [23,30]. However, upon SEPT9 depletion, not all filament assembly is blocked; instead, small curved septin filaments still form [30]. Consistent with previous reports of a role for SEPT9 in the late stages of cytokinesis [23], we found that depletion of SEPT9 by siRNA caused an increase in binucleate cells and an increase in the frequency of cells connected by ICBs (see electronic supplementary material, figure S5*a,b*). However, SEPT9 was not required for ICB formation as cells depleted of SEPT9 formed an ICB collar that extended and narrowed in the same manner as control cells (figure 3*c,d*) and SEPT11 still co-localized with anillin (figure 5*a,b*). Similarly, analysis of septin organization in SEPT9-depleted cells by 3D-SIM revealed filamentous arrays of septins and anillin (figure 5*c*). These data suggest that the formation of long helical septin filaments is not essential for ICB elongation.

### 3.5. Intercellular bridge tubule extrusion is dependent on septins

**Figure 6.**
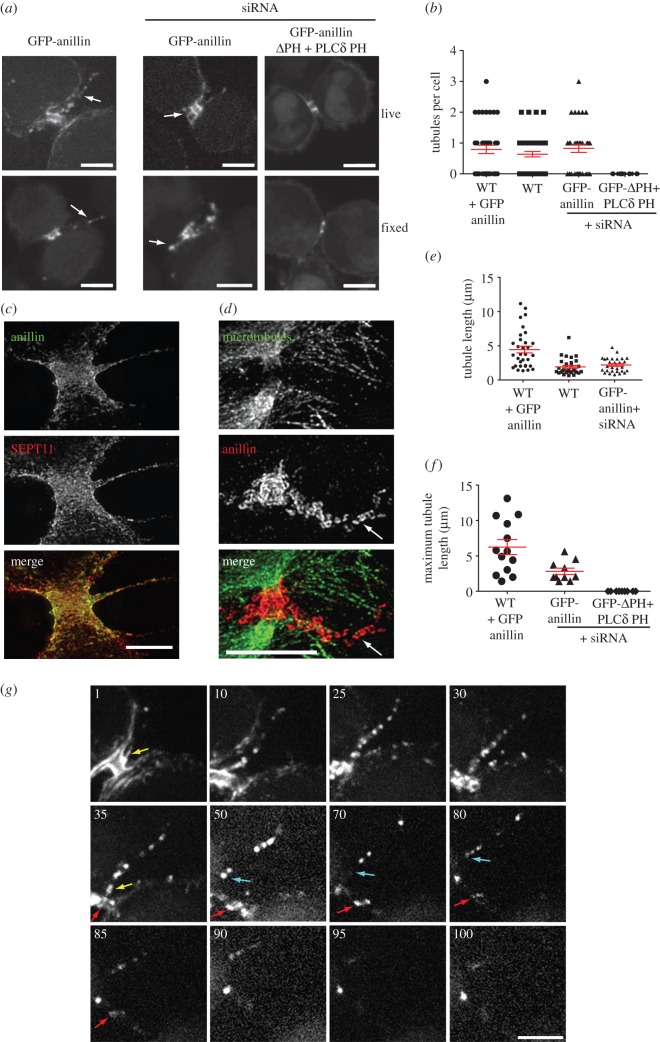
Tubules are extruded from ICBs during the elongation phase. (*a*) Images of tubules in HeLa cells expressing GFP-anillin or GFP-anillinΔPH + PLCδ PH from both TCA fixed and live samples. Arrows point to tubules. Scale bar, 5 μm. (*b*) Quantitation of the number of tubules observed in TCA fixed HeLa cells expressing different forms and levels of anillin. Overexpression of anillin (WT + GFP-anillin, *n* = 39), endogenous anillin (WT, *n* = 52), only GFP-anillin (*n* = 35) and only GFP-anillinΔPH + PLCδ PH (*n* = 32, respectively). (*c*) Subdiffraction microscopy of TCA fixed HeLa cells stained with anillin and SEPT11 antibodies showing an early stage ICB tubule. Scale bar, 5 μm. (*d*) Subdiffraction microscopy of TCA-fixed HeLa cells stained with anti-anillin and tubulin antibodies showing a late stage ICB tubule. Scale bar, 5 μm. (*e*) Tubule lengths in TCA-fixed HeLa cells expressing different forms and levels of anillin outlined in (*b*). (*f*) Quantification of the maximum observed length of ICB tubules during time-lapse imaging analysis of HeLa cell overexpressing GFP-anillin (WT + GFP-anillin, *n* = 11) or in the presence of siRNA expressing GFP-anillin (*n* = 9) or GFP-anillinΔPH + PLCδ PH (*n* = 11). (*g*) Time-lapse series of a HeLa cell expressing GFP-anillin focusing on the tubule extrusion phase. Numbers are minutes from an arbitrary starting point. Yellow arrow points to a tubule attached to the ICB. Blue arrow points to the same tubule that is now moving to one daughter cell. Red arrow marks the central of the three-anillin rings that defines the stem body. Scale bars, 5 μm.

Our time-lapse analysis of anillin dynamics during ICB elongation also revealed tubule-like flares of anillin extruding from the ICB during its elongation phase (figure 6*a* and the electronic supplementary material, video S10). In every cell observed live, at least one tubule was extruded per ICB. While most cells extruded a single tubule, in some cells up to three tubules were observed (figure 6*b*). The tubules contained both anillin and septins, but did not contain detectable tubulin or actin (see electronic supplementary material, figure S6). Intriguingly, the tubules were very transient, forming only during ICB elongation, suggesting they are involved in the early stages of ICB formation. While anillin tubules were prominent in live cell imaging experiments (maximum length of 2.82 ± 0.45 μm, *n* = 9, figure 6*f*), they could also be observed in fixed samples (figure 6*a–e*), being better preserved by trichloroacetic acid (TCA) fixation when compared with paraformaldehyde (PFA) or methanol fixation. The average tubule length was 1.93 ± 0.21 μm (*n* = 52) and 2.2 ± 0.2 μm (*n* = 35) in fixed mock-treated and GFP-anillin expressing cells, respectively (figure 6*e*). Tubule formation required anillin–septin interactions as no tubules were observed in cells expressing GFP-anillinΔPH-PLCδPH (figure 6*b*) and, conversely, 2.5-fold longer tubules were observed in cells overexpressing GFP-anillin (maximum length in live imaging experiments 7.06 ± 1.4 μm, *n* = 11, and average length in fixed cell analysis 4.45 ± 0.49 μm, *n* = 39, figure 6*e*,*f*).

During the lifetime of the tubule, the organization of anillin and septins changed. By using 3D-SIM, no apparent regular organization of anillin and septins was detected within the tubule in the early stages of tubule elongation (figure 6*c*). However, in longer tubules, emanating from more mature ICBs, distinct anillin–septin rings formed (figure 6*d*). These structures were dynamic and moved within the tubule (figure 6*g* and the electronic supplementary material, video S10). In addition, we found no evidence for tubules pinching off from the ICB; instead, tubules move to the daughter cell where they are reabsorbed into the cell body (figure 6*g* and the electronic supplementary material, video S10).

### 3.6. Septins are required for constriction site formation

**Figure 7.**
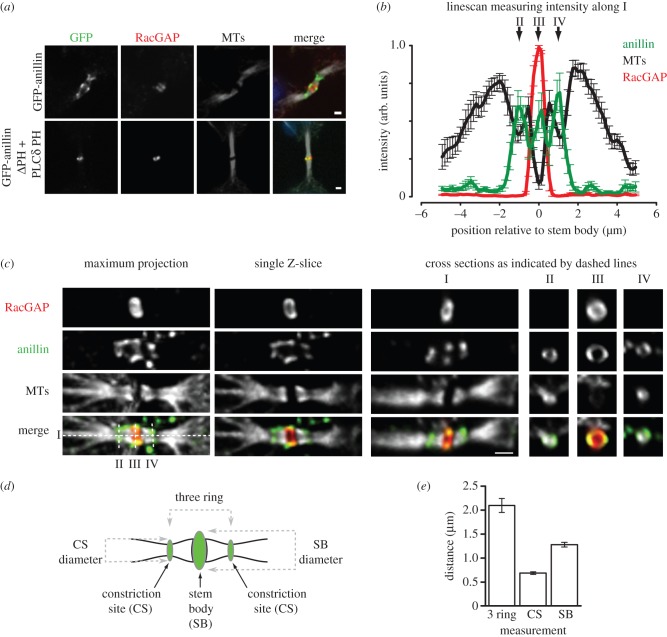
Anillin-dependent recruitment of septins to the ICB is required for constriction site ingression. (*a*) ICBs stained for microtubules and RacGAP in HeLa cells expressing GFP-anillin or GFP-anillinΔPH + PLCδ PH as the only forms of cellular anillin. Scale bar, 1 μm. (*b*) Quantification of fluorescence intensity along cross sections of ICBs (see I panel *c*) stained for tubulin (black), anillin (green) and RacGAP (red). *n* = 10. II, III and IV indicate the position of the cross sections in (*c*). (*c*) A ICB stained using antibodies to detect tubulin, anillin and RacGAP and viewed as a maximum projection and single *Z*-section in the *x-*, *y-* and *z*-planes as indicated. Scale bar, 1 μm. (*d*) Cartoon outlining the different ICB parameters measured in (*e*) and the spatial relationship of those rings to each other within the ICB. (*e*) Measurement of the diameter of the different rings in the three-ring anillin stage of ICB maturation.

Once the ICB had fully elongated, the collar of anillin and septins reorganized to generate three rings: a central ring at the stem body (1.28 ± 0.05 μm in diameter) flanked by two smaller rings (0.68 ± 0.03 μm in diameter) at the sites of microtubule constriction (figure 7). These measurements are consistent with previous studies that revealed regions of plasma membrane tightly constricted around microtubules [16,31]. RacGAP localized to the central ring, which must therefore be at the stem body (figure 7*a–c*). The targeting of anillin to the stem body was independent of septins, as GFP-anillinΔPH-PLCδPH also localized there (figure 7*a*). It is also noteworthy that the constriction sites may reflect sites of reduced microtubule density, as decreased tubulin fluorescence intensity was also observed there (figure 7*b,c*). Furthermore, as in previous studies [19], we found no anti-tubulin staining in the stem body, which is in contrast to GFP-tubulin fluorescence that decorates the stem body. After relocating to the sites of microtubule constriction, anillin was then gradually depleted from the ICB. This progressive loss of anillin was septin-dependent, as the non-septin-binding GFP-anillinΔPH-PLCδPH remained at the stem body throughout the rest of cytokinesis, failing to relocate to smaller ring structures flanking the stem body (figure 7*a*). Interestingly, the generation of the constriction site to which anillin is recruited is dependent upon septins.

### 3.7. ESCRT III recruitment to septin-dependent constrictions

**Figure 8.**
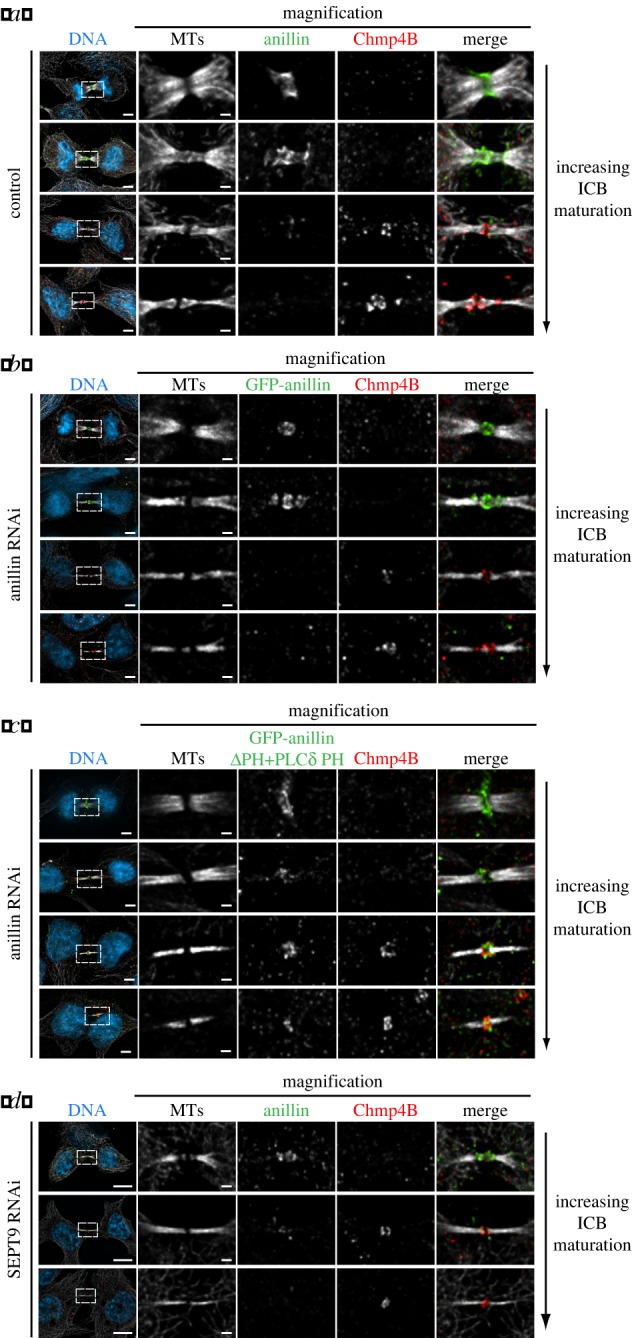
Anillin-dependent recruitment of septins to the ICB is required for Chmp4B localization to the abscission site. (*a*) Images of ICBs with an increasing degree of maturation stained for anillin, tubulin and Chmp4B. (*a*) Images of ICBs with an increasing degree of maturation expressing GFP-anillin as the only form of anillin and stained for tubulin and Chmp4B. (*c*) Images of ICBs with an increasing degree of maturation expressing GFP-anillinΔPH + PLCδ PH as the only form of anillin and stained for tubulin and Chmp4B. (*d*) Images of ICBs with an increasing degree of maturation in HeLa cells depleted of SEPT9 stained for anillin, tubulin and Chmp4B. Bar in whole cell images, left-hand side is 5 μm, Bar in magnified images is 1 μm.

The final stage of cytokinesis requires the recruitment of the abscission machinery to the ICB to facilitate the physical separation of the daughter cells. A primary component of the abscission machinery is the ESCRT III complex, which promotes further constriction of the ICB for the abscission event [14]. We established that Chmp4B is targeted to the stem body after anillin and septins have been removed from the ICB (figures 2*e* and 8*a,b*), suggesting that anillin does not have a direct role in abscission. However, building and organizing an ICB may be a prerequisite for abscission. To test this model, we analysed the role of anillin and septins in establishing the abscission site. In cells only expressing GFP-anillinΔPH-PLCδPH, Chmp4B was still targeted to the GFP-anillinΔPH-PLCδPH positive stem body (figure 8*c*), suggesting that septins are required for the timely removal of anillin from the ICB. Although Chmp4B was targeted to the stem body in GFP-anillinΔPH-PLCδPH expressing cells, Chmp4B remained at the stem body for the duration of cytokinesis. This defect in Chmp4B relocalization to an abscission site probably explains the abortive cytokinesis in cells expressing GFP-anillinΔPH-PLCδPH resulting in binucleate cells. As SEPT9 has been implicated in the very late stages of cytokinesis [23], we assessed the role of SEPT9 in Chmp4B targeting to the abscission site. In cells depleted of SEPT9 by siRNA, Chmp4B localized to the stem body but did not relocalize late in cytokinesis to the abscission site (figure 8*d* and the electronic supplementary material, figure S5*c,d*). In contrast, upon SEPT9 depletion, anillin still localized to the stem body and relocalized to the constriction site (see electronic supplementary material, figure S5*d*). The failure of Chmp4B to relocalize to constriction sites in the absence of SEPT9 is reminiscent of Chmp4B dynamics in cells expressing GFP-anillinΔPH-PLCδPH that do not recruit septins to the ICB, suggesting that an important role of anillin during the late stages of cytokinesis is to facilitate SEPT9 recruitment to the ICB. These data suggest that the final role of the anillin–septin cytoskeleton is to constrict the ICB to prime it for the recruitment of ESCRT III to the future site of abscission.

## 4. Discussion

Cytokinesis is primarily a process of membrane remodelling involving a series of steps that ingress the plasma membrane between the segregating chromosomes, and culminating in abscission to release the two daughter cells from each other. By examining anillin dynamics and using a novel variant that fails to recruit septins to the cytokinetic machinery, but allows the early stages of cytokinesis to proceed, we have established new roles for the anillin–septin cytoskeleton in the formation of the ICB and the generation of the site of abscission.

### 4.1. Anillin–septin filaments promote intercellular bridge elongation

Using high-resolution 3D-SIM, we were able to observe anillin–septin filaments, for the first time, in the ICB. Filaments could be seen throughout the ICB during its elongation phase. The filaments run around the inner face of the plasma membrane of the ICB, perpendicular to the long axis of the ICB. Our works extend previous images of anillin taken in *Caenorhabditis elegans* during polar body extrusion that suggested rings of anillin formed during this process [32]. In addition, our high-resolution images are consistent with filaments forming either a continuous helix or a series of stacked rings. We favour the latter possibility because depletion of SEPT9, which is required for long interphase septin filament assembly but still allows the formation of short, curved filaments [30], affected neither septin filament formation in the ICB nor ICB elongation. Therefore, our data more readily support a model whereby the formation of short, curved septin filaments, possibly stacked upon each other, is a more likely mechanism for driving ICB elongation. However, as depletion of SEPT9 disrupts the formation of the abscission site, we cannot rule out the possibility that long helical septin filaments play some role in abscission site assembly.

Previous electron microscopy work has described ripples on the plasma membrane of the ICB that could be explained by the presence of filaments just beneath [14,31]. In the study of Mullins and Biesele [31], the ripples were seen on the arms of early stage ICBs. By contrast, Guizetti and co-workers [14] described ripples and filaments going from the stem body to the abscission site in more mature ICBs. These 17 nm filaments were proposed to be formed by ESCRT III components, as ESCRT III has been reported to form filaments. In other cellular contexts, ESCRT III filaments are restricted to the neck of membrane tubes with very narrow diameters, indeed much narrower than that of the ICB directly surrounding the stem body. As we do not detect anillin or septins at the very narrow final abscission site, it is probable that the filaments previously observed in the mature ICBs emanating from the stem body to the abscission site are ESCRT III filaments [14]. Our temporal analysis finds that ESCRT III, as defined by Chmp4B localization, does not localize to ICBs in early stage ICBs, suggesting that the membrane ripples observed in the study of Mullins and Biesele are unlikely to be caused by Chmp4B. Rather, in pre-abscission phase ICBs comparable with those described in the Mullins and Biesele study, we clearly observe anillin–septin filaments along the arms of the ICBs, leading us to speculate that these filaments could cause the observed ripples. Combined, these studies suggest that multiple filamentous arrays are involved in the final stages of cytokinesis: anillin–septin filaments driving both ICB formation and constriction site initiation and ESCRT III filaments driving abscission.

Interestingly, the failure to recruit septins to the ICB causes a long pause in cytokinesis where the length the ICB ring is equal to its diameter. In *Dictyostelium*, such a point in cytokinesis has been proposed to be an important transition point, *D_x_*_,_ where forces of the contractile ring balance the elastic properties of the cortex [33,34]. Our data suggest that septins are involved in driving the cell through this transition point to the next stage of cytokinesis.

### 4.2. Tubule extrusion from intercellular bridges

During ICB formation, we observed the extrusion of membrane tubules from the surface of the ICB. How tubules are extruded from the surface of the ICB remains unclear. As we observe no tubules in the absence of septin recruitment, it is tempting to speculate that septins stimulate tubule extrusion. Septins can tubulate liposomes *in vitro* [35]; however, in these assays, septins are on the outside of the liposome ‘pulling’ out the membrane tubule. By contrast, septins are on the inside of the ICB tubules, a localization that would require septins to ‘push’ the tubule out. Currently, evidence supporting this mode of action for septins is lacking. Alternatively, septins could stabilize ICB tubules that have been generated by other factors. Such a function has been proposed for septins in cleavage furrow ingression during cellularization in syncytial *Drosophila* embryos [36].

Once extruded during the elongation phase, the tubules then migrate along the ICB to one of the daughter cells where they are subsequently reabsorbed back into the plasma membrane of the cell body. How this process occurs remains to be determined. However, the existence of such a mechanism does allow for the contents of the tubules to be recycled rather than lost, as would occur if the tubules were pinched off.

Tubules have been previously observed in many electron microscopy studies (including, but not limited to Mullins & Biesele [31] and Schroeder [37]). More recently, ICB tubules in neuroepithelial cells have been observed that contain tubulin and are shed from the ICB [38]. The tubules from neuroepithelial cells would therefore appear to be different from those we observe that lack tubulin and which do not appear to be shed from the ICB. Much of the previous work has focused on membrane reorganization events that occur within the ICB. However, our studies combined with those above suggest that there may be considerably more membrane reorganization than may have previously been appreciated, particularly as different classes of tubules appear to be extruded from the ICB during its maturation. Membrane tubules have also been described protruding from the tip of the cleavage furrows during cellularization in *Drosophila* embryos, a structure that bears many similarities to the cytokinetic furrow [39,40]. Likewise, these tubules are not homogeneous, being defined by different components [40]. While the precise role of these distinct types of tubules has not been defined, their presence or absence correlates with changes in membrane ingression dynamics where they may act to regulate the availability of membrane for furrow assembly [40]. Therefore, we propose that tubulation of the plasma membrane during ICB biogenesis may allow for the rapid, site-specific removal of plasma membrane that could not be achieved through the endocytosis of vesicles into the lumen of an increasingly dense ICB. The removal of large amounts of plasma membrane may act like the cords of a purse in that when the cords are pulled, the open neck narrows and closes in response. Likewise, the extruded tubules would draw plasma membrane from the elongating ICB, thereby tightening the remaining plasma membrane around the bundled microtubules, suggesting the ICB is actively rather than passively generated.

### 4.3. Septin-mediated recruitment of anillin prepares the abscission site

As cytokinesis progresses, anillin and septins localize to three discrete rings within the ICB. Intriguingly, two of the rings are of a narrower diameter and form on either side of the stem body. At these sites, microtubules appear less dense and more constricted. While the precise function of the two rings flanking the stem body remains to be determined, it is possible that one of these sites matures to form the abscission site. What is clear is that septins play fundamental roles in abscission site formation. When septins are not recruited to the ICB, no constriction sites are formed, and the relocalization of the ESCRT III component Chmp4B does not occur. Such observations are consistent with the model that the biogenesis of the future abscission site must be formed prior to Chmp4B recruitment and the subsequent abscission [15]. Furthermore, they suggest that septins are required for constriction site formation. These findings raise important questions about the order of events that lead to abscission. In contrast to the model of ESCRT III solely driving membrane ingression by the formation of a continuous filamentous array initiated at the stem body, our data and that of others [15,16] suggest that the ingression that leads to abscission is more complex: the anillin–septin cytoskeleton first makes an initial ingression coincident with localized microtubule reorganization, after which ESCRT III components are recruited to the ingression site and drive further constriction leading to abscission and the release of the daughter cells from each other. Cytokinesis is therefore achieved by the sequential action of different contractile machines that are dependent upon each other's actions in order to complete cytokinesis (see electronic supplementary material, figure S7). The actomyosin cytoskeleton initiates the large-scale ingression of the cleavage furrow over tens of micrometres, then anillin–septins thin the ICB and constrict microtubules to ingress the ICB by hundreds of nanometres, and finally the ESCRT III complex pinches the ICB and physically separates the daughter cells.

## 5. Material and methods

### 5.1. Cell culture

HeLa cells were cultured in Dulbecco's modified Eagle's medium (DMEM3; Sigma) supplemented with 10% fetal bovine serum (FBS) (Invitrogen) and 1% penicillin/streptomycin (Invitrogen) in an air–5% CO_2_ atmosphere at 37°C with constant humidity.

### 5.2. Generation and characterization of stable cell lines

Generation of stable cell lines expressing eGFP-anillin and eGFP-anillinΔPH-PLCδPH from a Tet-inducible promoter was done using the Flp-In system (Invitrogen). Full-length anillin and anillinΔPH-PLCδPH [10] were cloned into pcDNA5/FRT/TO/eGFP-N vector to generate N-terminally tagged eGFP constructs. Cell lines were then generated as per manufacturer's instructions. Transfection of a HeLa Flp-In host cell line containing an integrated FRT site and expressing the tetracycline repressor protein (TetR) was done using lipofectamine 2000, and cell lines were selected for using 200 μg ml^−1^ hygromycin B (Bioshop). Expression of TetR was maintained using 5 µg ml^−1^ blasticidin (Invitrogen). Individual colonies were expanded and screened for the presence of GFP by immunostaining and western blotting with an anillin antibody. The resulting cell lines, which inducibly express GFP-anillin or GFP-anillinΔPH-PLCδPH, were maintained in DMEM with 10% FBS, 1% penicillin/streptomycin, 5 µg ml^−1^ blasticidin and 200 µg ml^−1^ hygromycin. Expression of the GFP tagged constructs was induced with 1 µg ml^−1^ doxycycline (Bio Basic). Overexpression experiments were performed after treating the cell with 1 µg ml^−1^ doxycycline for 24 h prior to analysis.

### 5.3. Western blotting

Western blotting was performed according to standard procedures using the following primary antibodies: anillin (Santa Cruz Biotechnology, Inc.), and SEPT 9 and SEPT11 [41] (gifts from W. Trimble, University of Toronto).

### 5.4. siRNA treatment and rescue

HeLa cells were grown to 50–60% confluency and transfected with 40 nM or 120 nM double-stranded siRNA (anillin and SEPT9, respectively) using lipofectamine 2000 (Invitrogen). For rescue experiments, GFP-anillin or GFP-anillinΔPH-PLCδPH was induced for 2 h, 16 h after siRNA transfection; live cell imaging or cell fixation was then carried out 24–30 h after siRNA transfection. SEPT9 depletion was achieved 56 h after transfection. All siRNAs were obtained from Integrated DNA Technologies. 3′-UTR siRNA duplexes for anillin (5′-agcuuacagacuuagcau-3′) and the negative control siRNA (5′-cguuaaucgcguauaauacgcgut-3′) were previously used in Liu *et al.* [10]. siRNA used to deplete SEPT9 (5′-gcacgauauugaggagaaa-3′) was based on that described in Estey *et al*. [23].

### 5.5. Immunofluorescence

Cells cultured on glass coverslips thickness no. 1½, size 22 × 22 mm (Electron Microscopy Services) were stained by standard methods after fixation with 10% TCA–CBS, 4% PFA or 100% methanol (see the electronic supplementary material, table S1 for antibody information), performed as follows.

For 10% TCA–CBS fixation (adapted from Hayashi *et al.* [42]), ice-cold 10% trichloroacetic acid (Bioshop) buffered in cytoskeleton buffer + sucrose (10 mM MES pH 6.1, 138 mM KCl, 3 mM MgCl_2_, 2 mM EGTA, 0.32 M sucrose) was added to cells and incubated for 15 min on ice. Cells were permeabilized with 0.2% Triton X-100, 50 mM glycine in phosphate-buffered solution (PBS) on ice for 2 min and quenched with 50 mM glycine in PBS for 20 min. For 4% PFA fixation, cells were fixed in 4% paraformaldehyde (EMD) in PBS for 20 min at room temperature; cells were then permeabilized in 0.1% Triton in PBS for 5 min at room temperature. For 100% methanol fixation, methanol (Caledon) was chilled to −20°C, added to cells and incubated at −20°C for 20 min.

Secondary antibodies used were goat or donkey anti-mouse/rabbit/goat conjugated to Alexa Fluor-488, -594 or -647 (Invitrogen). Far-red phalloidin (Invitrogen) was used to visualize actin in 4% PFA fixed cells. In addition cells were counterstained with 4′,6-diamidino-2-phenylindole (DAPI, Roche) to observe DNA. Coverslips were then mounted onto glass slides (VWR) with Mowiol (polyvinyl alcohol 4-88, Fluka) and imaged using a Nikon TE2000 inverted confocal spinning disc microscope with a 60×/1.4 NA oil-immersion objective lens and 1.515 immersion oil (Nikon) at room temperature. Images were acquired using Metamorph software (Molecular Devices) driving an electron multiplying charge-coupled device (CCD) camera (ImagEM, Hammamatsu). Z sections (0.2 µm apart) were acquired to produce a stack that was then imported into AutoQuant X2 (Media Cybernetics) for deconvolution (10 iterations). Maximum projections and cross sections were performed using Metamorph. Images were overlaid in Photoshop (Adobe), involving adjustments in brightness and contrast of images.

### 5.6. Time-lapse microscopy

Cells cultured on circular glass coverslips, thickness no. 1, diameter 25 mm (Fisher Scientific) were treated as indicated and mounted in a heated chamber containing air–5% CO_2_ atmosphere at 37°C (Live Cell Instrument Systems) in dye-free DMEM with 10% FBS (Invitrogen) mounted on a Nikon TE2000 inverted microscope equipped with a spinning disc confocal scanning head driven by Metamorph software as described above. Time-lapse video microscopy was used to follow cells with a stack of images (*z*-step 2 µm) taken every 1 min using a 40×/1.0 NA PlanApo oil-immersion objective lens and 1.515 immersion oil (Nikon).

### 5.7. Subdiffraction three-dimensional structured illumination microscopy

HeLa cells were grown on glass coverslips, thickness no. 1½, size 22 × 22 mm. Following 10% TCA–CBS fixation, cells were stained and coverslips were mounted onto glass slides with ProLong Gold antifade mounting medium (Invitrogen). Secondary antibodies used were donkey anti-mouse/rabbit/goat conjugated to Alexa Fluor-488 or Alexa Fluor-594 (Invitrogen). Super-resolution microscopy imaging was performed as described previously [29]. Images were acquired sequentially using a three-dimensional structured illumination microscope (OMX v3, Applied Precision) equipped with 405, 488 and 592.5 nm diode lasers, electron multiplying CCD cameras (Cascade II 512 × 512, Photometrics), and a 100×/1.40 NA PlanApochromat oil-immersion objective lens (Olympus) at room temperature. 3D-SIM image stacks were reconstructed and aligned using the softWoRx 5.0 software package (Applied Precision) with the following settings: pixel size 39.5 nm; channel-specific optical transfer functions; Wiener filter 0.002; discarding negative intensities; default value of 65 for background intensity; drift correction with respect to first angle; and custom K0 guess angles for camera positions. Images were rendered using AutoQuant X2 software to generate three-dimensional images and movies exported showing three-dimensional rotations (±30° around *y*-axis, five frames per second). Maximum projections and cross sections were done using Metamorph. Images were overlaid in Photoshop (Adobe), involving adjustments in brightness and contrast of images.

### 5.8. Fluorescence recovery after photobleaching

Experiments were performed at 37°C in a heated chamber containing air–5% CO_2_ atmosphere at 37°C (Live Cell Instrument Systems) in dye-free DMEM with 10% FBS (Invitrogen) using a Nikon A1R laser scanning confocal microscope equipped with high-QE Hamamatsu photomultiplier, driven by NIS-Elements software v. 4.0 (Nikon) using a 60×/1.4 oil-immersion lens and 1.515 immersion oil (Nikon). After an initial series of images collected with the laser at 4% full power (pre-bleach), a defined region of the cell was photobleached with the laser at full power. Subsequent images, taken at 5 s intervals with the laser at 4% full power, were obtained in order to follow the recovery of the fluorescence signal within the bleached region. Fluorescence quantification was performed using ImageJ software (NIH). Background levels were measured from a non-fluorescent region and subtracted from both experimental and reference regions of interest. The corrected intensity was then normalized relative to an unbleached reference region. The fluorescence intensity was presented as a percentage of the averaged pre-bleach levels. Fluorescence recovery was calculated by nonlinear (one phase association) regression curve fit using GraphPad Prism software.

### 5.9. Intercellular bridge staging

Time-lapse video microscopy of cells expressing GFP-tubulin was used to observe microtubule ICB maturation. A stack of images (*z*-step 0.6 µm) was acquired every 5 min using a 40×/1.0 NA PlanApo oil-immersion objective lens, at 37°C. Images were then deconvolved, and images were segmented based on an exclusive threshold, with the grey values above the threshold limit of 98% of the intensity values. ICB formation and maturation were quantified by width of bundled microtubules as identified by the threshold; the width at both ends of each ICB for every time point was measured and averaged to determine width of ICB microtubules (independent of stem body and constriction sites) as the cell progressed through the final stages of cytokinesis up to the timepoint before abscission. For fixed cells, images were acquired and deconvolved as before and thresholding and microtubule ICB width measurements were done as for live cells. Each ICB was then grouped based on the anillin localization as follows: ‘furrow’ anillin, concentrated on a curved membrane; ‘collar’ anillin, uniform, parallel along microtubule bundle; ‘three ring’ anillin, intensity formed three peaks along the ICB; ‘dissipation’ anillin, localization very faint/dissipated from ICB region.

### 5.10. Intercellular bridge, furrow and collar measurements

Images of ICBs at various stages were acquired. Intensity profiles were measured by longitudinal linescans (line width = 3 pixels) along maximum projection deconvolved images using Metamorph. ICBs at the three-ring stage were identified by the presence of three distinct anillin intensity peaks. Intensity values were normalized relative to the maximum value for each plot. Profiles of ICBs at the three-ring stage were then overlaid relative to the central RacGAP peak. Distances were measured as the distances between peaks, as indicated in text and figures. Diameter measurements were done from a single *z*-section at the ICB centre at indicated positions.

Furrow width was measured by drawing a line from one tip of furrow to the other tip of furrow using single plane images. Rates of furrow ingression were calculated by linear regression (absolute value of slope) of the slow initial phase (maximum width to 90%) and the fast ingression phase (90–15% maximum width). Duration of the initial slow phase was calculated as the time required to reach 90% maximum width from anaphase onset. Collar length was measured by drawing lines along both sides of collar and average numbers were used. Collar width was measured as the distances between two peaks using line scan.

### 5.11. Co-immunoprecipitation assay

To assay the ability of wild-type anillin and anillinΔPH-PLCδPH to bind to septins, HeLa cell lines stably expressing GFP-anillin and GFP-anillinΔPH-PLCδPH were synchronized using a double thymidine block and then released for 12 h to enrich for late mitotic cells. Cells were harvested by incubating with PBS + 2 mM EDTA at 4°C, centrifuged and the cell pellet was lysed in ice-cold non-denaturing lysis buffer (1% (w/v) Triton X-100, 150 mM NaCl, 10 mM sodium phosphate pH 7.2, 2 mM EDTA, 50 mM NaF) with 1% (v/v) protease inhibitor cocktail (Roche). Lysate (500 μl) was then incubated with 5 μg anti-GFP antibody (Roche) and 40 μl protein G sepharose 4 Fast Flow (GE Healthcare) for 16 h at 4°C with rotation. Protein G sepharose was washed four time with lysis buffer, boiled in SDS sample buffer and analysed by Coomassie staining and western blot with anti-anillin and anti-SEPT11 antibodies.

### 5.12. Statistical analysis

All experiments were repeated at least three times, and statistical analyses were conducted using Prism (GraphPad). Student's *t*-tests and Fisher's exact tests were used to determine statistical significance of continuous and nominal (contingency table) data, respectively. A *p*-value of < 0.05 was considered statistically significant.

## Supplementary Material

Supplementary Figure S1

## Supplementary Material

Supplementary Figure S2

## Supplementary Material

Supplementary Figure S3

## Supplementary Material

Supplementary Figure S4

## Supplementary Material

Supplementary Figure S5

## Supplementary Material

Supplementary Figure S6

## Supplementary Material

Supplementary Figure S7

## Supplementary Material

Supplementary Table 1
