# Supplementary figures and images for: Anillin-dependent organization of septin filaments promotes intercellular bridge elongation and Chmp4B targeting to the abscission site
Source: Open Biol. 2014 Jan 22;4(1):130190. doi: 10.1098/rsob.130190 (PMC3909275; doi:10.1098/rsob.130190)

## Supplementary Figure 1

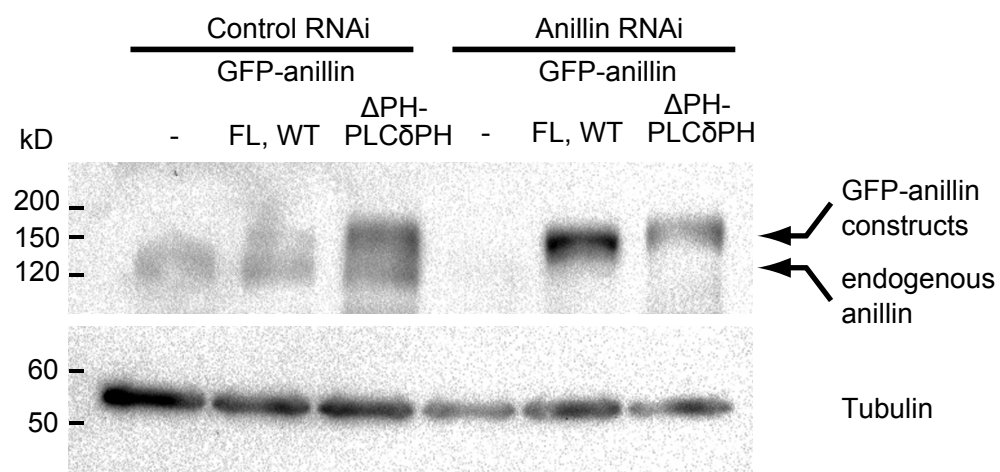

Supplement: Supplementary Figure S1 [file rsob130190supp1.pdf]

Supplementary Figure S2

A

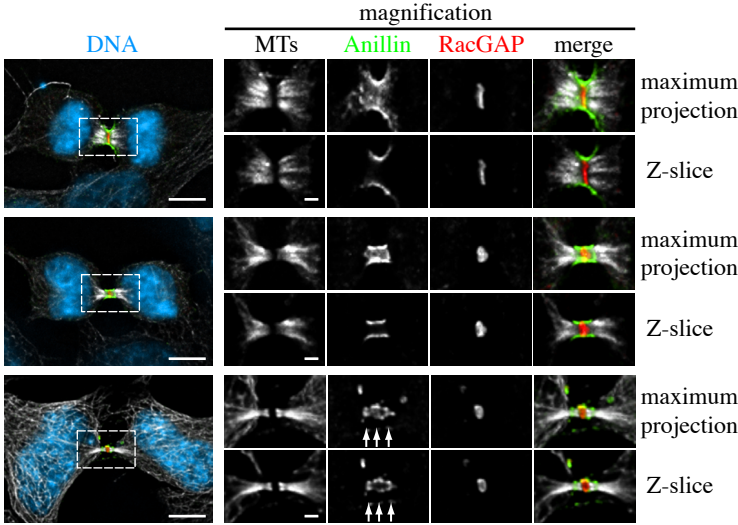

B

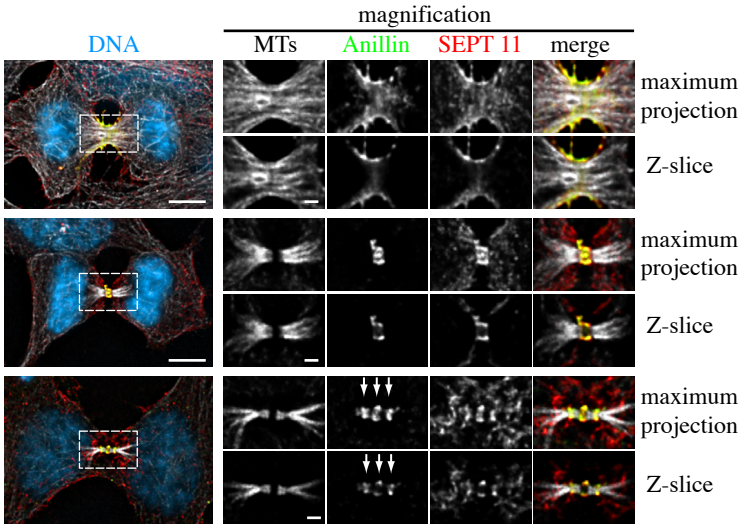

C

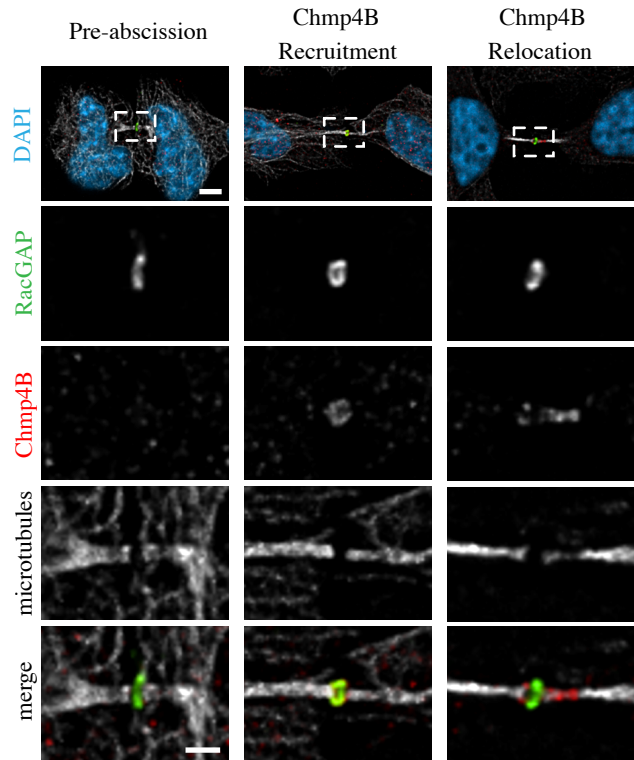

Supplement: Supplementary Figure S2 [file rsob130190supp2.pdf]

Supplementary Figure S3

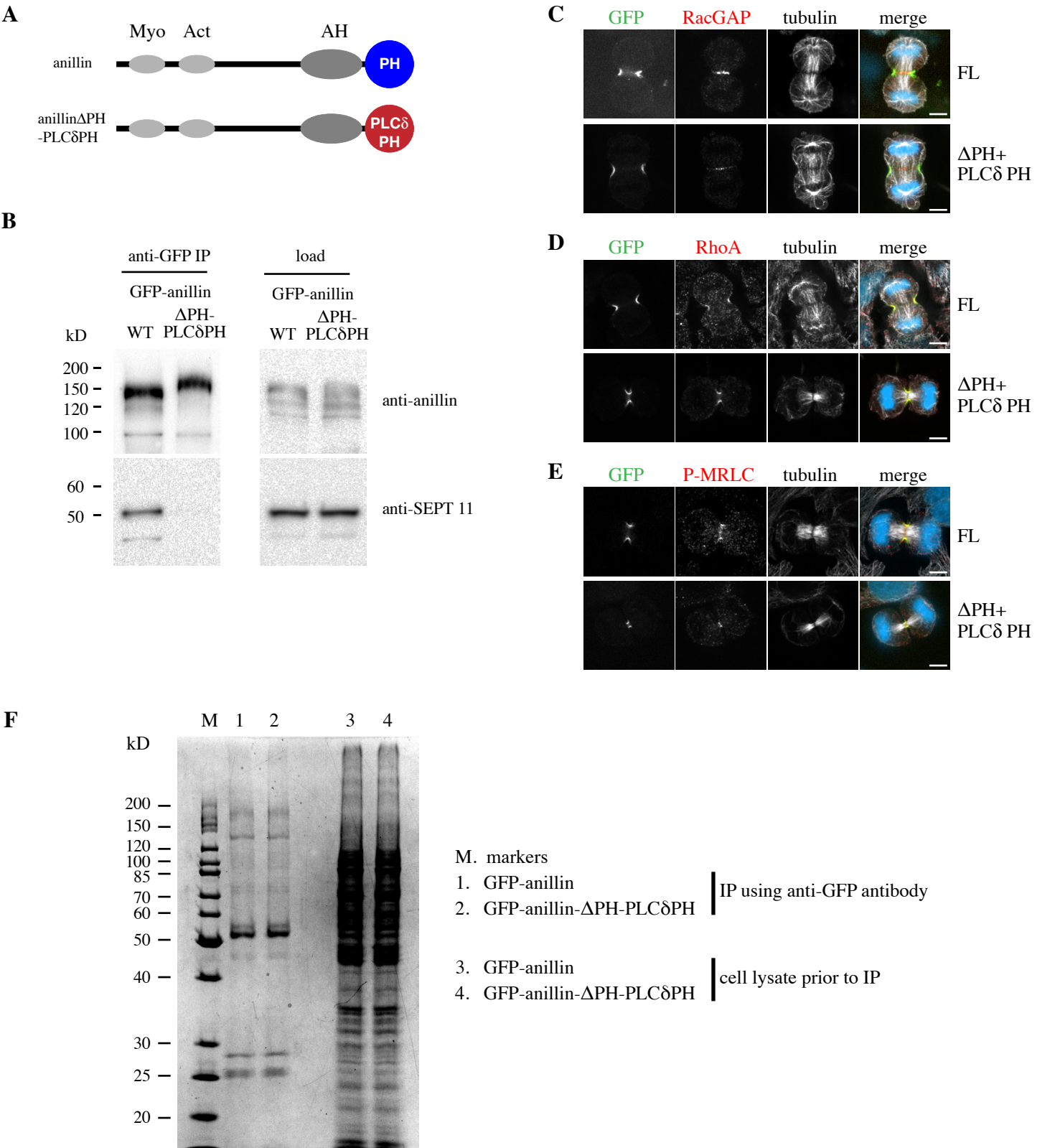

Supplement: Supplementary Figure S3 [file rsob130190supp3.pdf]

Supplementary Figure S4

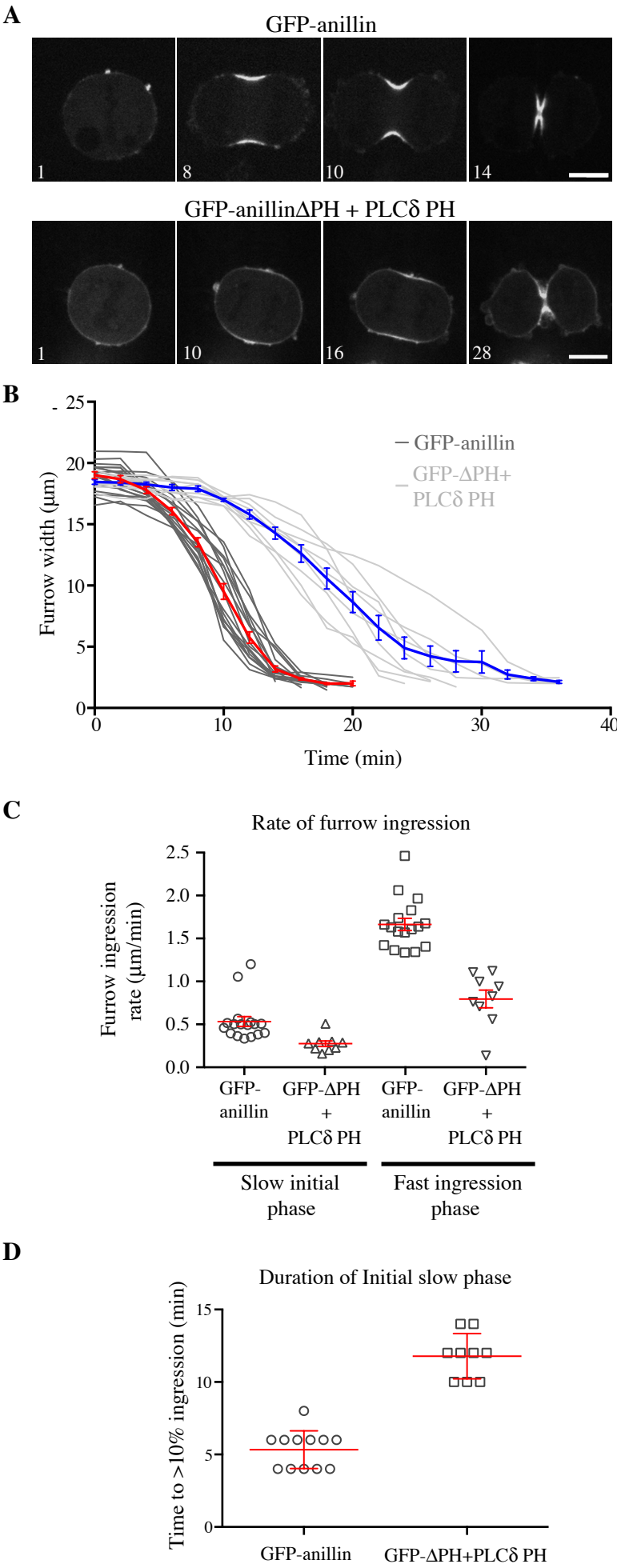

Supplement: Supplementary Figure S4 [file rsob130190supp4.pdf]

Supplementary Fig S5

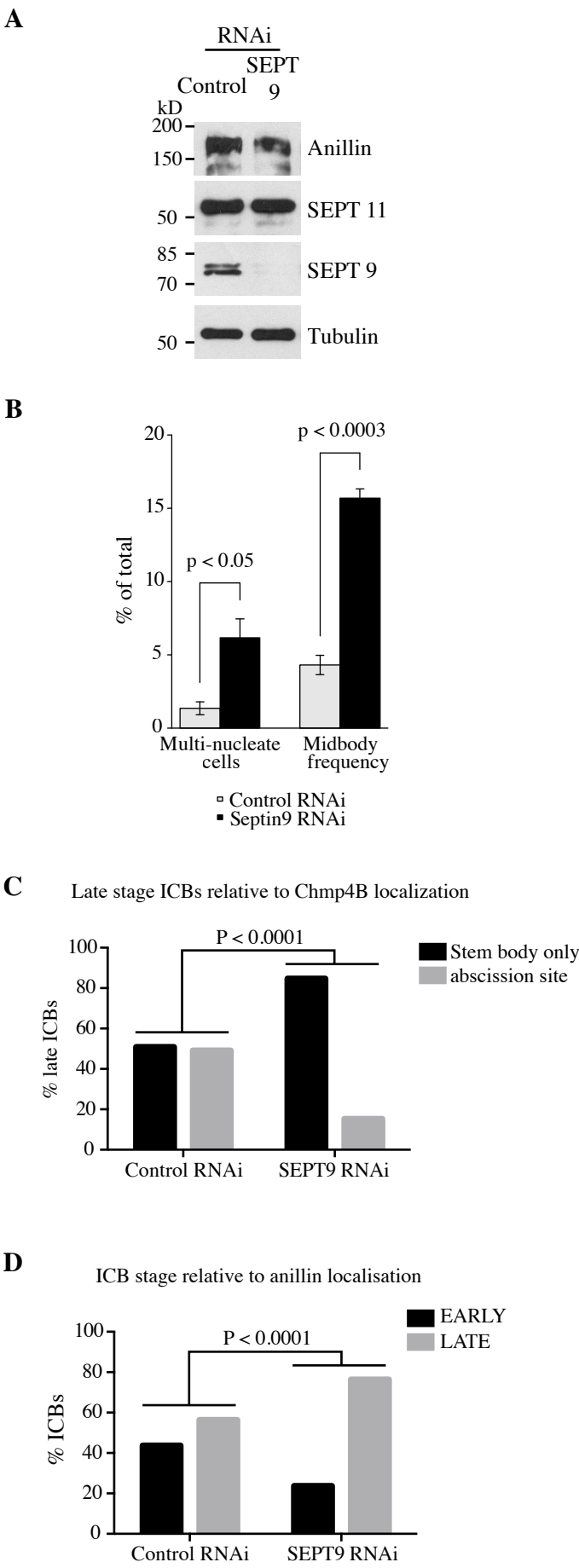

Supplement: Supplementary Figure S5 [file rsob130190supp5.pdf]

**Supplementary Figure S6**

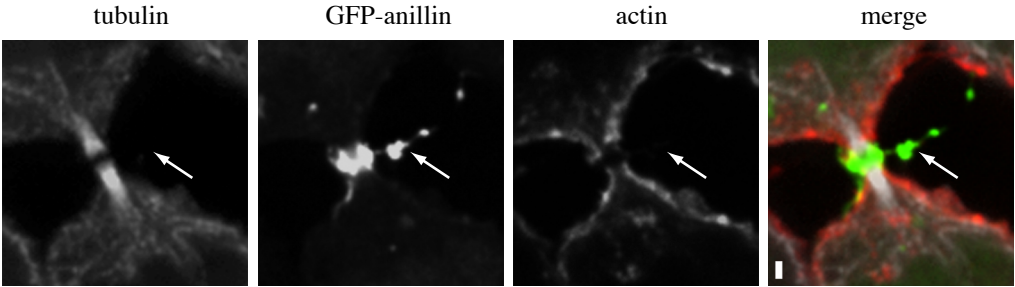

Supplement: Supplementary Figure S6 [file rsob130190supp6.pdf]

Supplementary Figure S7

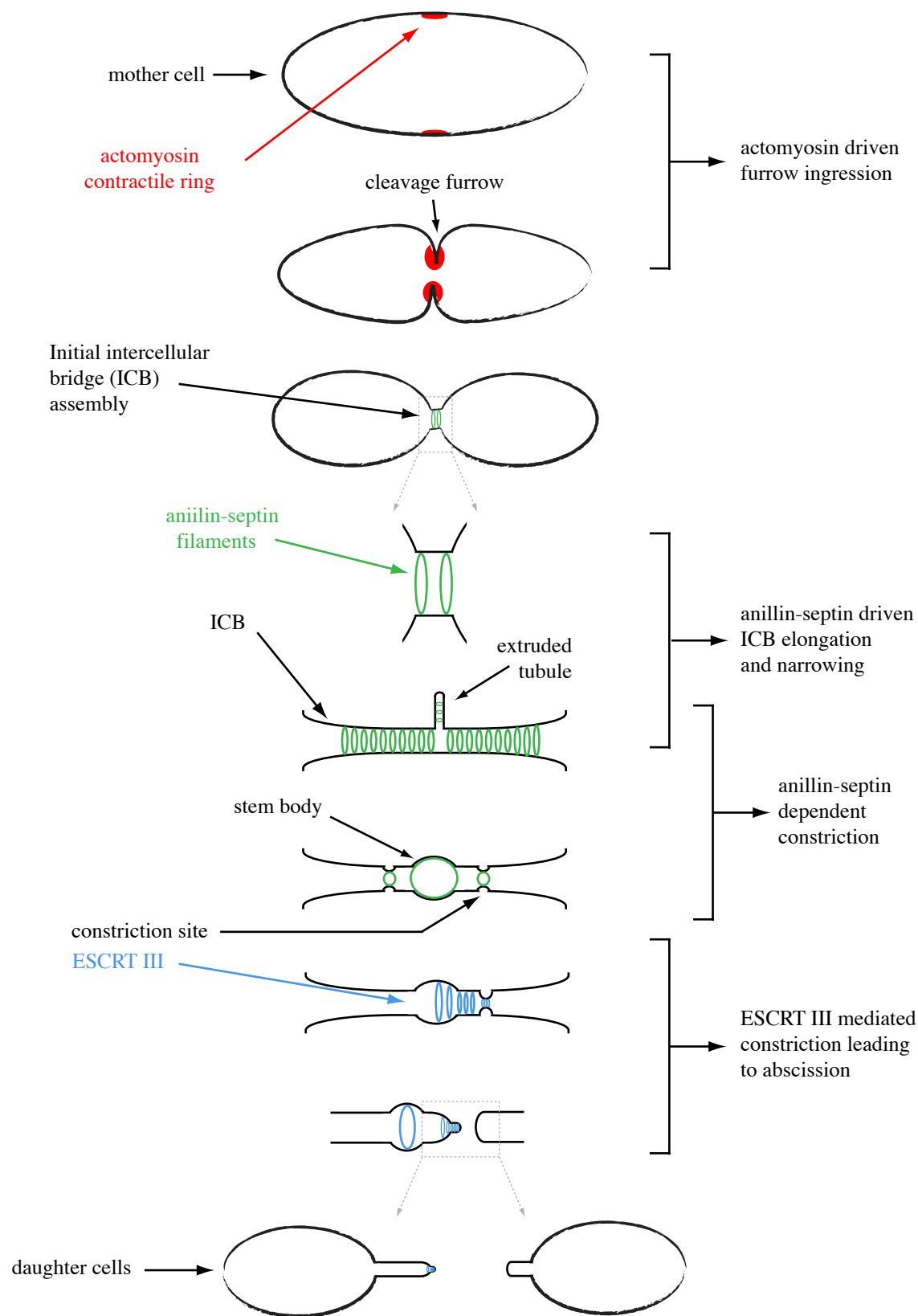

Supplement: Supplementary Figure S7 [file rsob130190supp7.pdf]
