# Supplementary material for: Anillin-dependent organization of septin filaments promotes intercellular bridge elongation and Chmp4B targeting to the abscission site
Source: Open Biol. 2014 Jan 22;4(1):130190. doi: 10.1098/rsob.130190 (PMC3909275; doi:10.1098/rsob.130190)
Supplement: Supplementary Table 1 [file rsob130190supp8.doc]

|  | | | | | Fixation method | | |
| --- | --- | --- | --- | --- | --- | --- | --- |
|  | Anti | Species | Company/Source | Ref | TCA | PFA | Methanol |
| pAb | Anillin | Rabbit | Santa Cruz | sc-67327 | ✓✓ | ✓ | ✓ |
| pAb | Anillin | Goat | Santa Cruz | sc-54859 | ✓✓ | ✓ | ✓ |
| pAb | Septin 11 | Rabbit | Trimble Lab | Huang et al., 2008 | ✓✓ | ✓ | ✓ |
| mAb | alpha Tubulin | Mouse | Sigma | DM1a | ✓✓ | ✓ | ✓✓ |
| pAb | alpha Tubulin | Rabbit | AbCam | ab18251 | ✓✓ | ✓✓ | ✓✓ |
| pAb | Chmp4B | Rabbit | Santa Cruz | sc-82557 | 🗶 | 🗶 | ✓ |
| pAb | RacGAP | Goat | Novus Biologicals | NB 100-884 | ✓✓ | ✓ | ✓✓ |
| mAb | RhoA | Mouse | Santa Cruz | sc-418 | ✓✓ | 🗶 | 🗶 |
| pAb | P-Myosin Light Chain (S19) | Rabbit | Cell Signalling Technology | 3671 | 🗶 | ✓✓ | 🗶 |

**Supplementary Table 1**

Primary antibodies used for immunofluorescence. pAb = polyclonal antibody, mAb = monoclonal antibody. Fixation conditions for immunofluorescence: ✓✓ - Excellent ✓ - Satisfactory 🗶 - poor
